# Preferences and Avoidance of Sleeping Positions Among Patients With Chronic Low Back Pain: A Cross-Sectional Study

**DOI:** 10.7759/cureus.59772

**Published:** 2024-05-06

**Authors:** Jari Ylinen, Arja Häkkinen, Hannu Kautiainen, Juhani Multanen

**Affiliations:** 1 Physical Therapy, Nova, Central Hospital of Central Finland, Jyväskylä, FIN; 2 Faculty of Sport and Health Sciences, University of Jyväskylä, Jyväskylä, FIN; 3 Unit of Primary Health Care, University of Kuopio, Kuopio, FIN; 4 Health and Welfare, South-Eastern Finland University of Applied Sciences, Savonlinna, FIN

**Keywords:** working-age population, epidemiologic studies, sleeping habits, oswestry index, disability, sleeping posture, sleeping ergonomics

## Abstract

Background

Chronic low back pain (CLBP) is a common issue among the working-age population. Sleeping position may affect CLBP, with the prone position commonly suggested to be avoided. This study aims to examine the relationship between preferred and avoided sleeping positions and to explore the frequency of increased pain in various sleeping positions among patients with nonspecific CLBP and pain and disability levels.

Methods

This cross-sectional study included all adult patients referred for specialist consultation for CLBP at the outpatient clinic of the Central Hospital of Central Finland’s spine department. Pain intensity was measured using a visual analog scale (VAS), and disability was assessed with the Oswestry Disability Index (ODI). Patients completed a questionnaire detailing the main sleeping positions and positions avoided due to low back pain (LBP).

Results

The study enrolled 375 consecutive patients, with a mean age of 51 ± 17 years; 64% (n=240) were female. The mean VAS score was 63 ± 24, and the mean Oswestry Index was 38 ± 18%. The majority of patients (87%, n=327) reported sleeping in a side-lying position, followed by supine (47%, n=176) and prone (22%, n=82) positions. A negative correlation was found between age and the preference for sleeping in the prone position. No significant gender differences in sleep positions were observed (p=0.69). Sleep was disturbed in 77% of patients (n=289) due to LBP, and 87% (n=327) reported difficulties due to LBP when getting up. Overall, 92% of participants (n=345) experienced difficulties sleeping or getting up in the morning due to LBP. Many patients avoided certain positions due to pain: 42% (n=157) avoided the prone position, 35% (n=131) the back, 15% (n=56) the left side, and 13% (n=49) the right side. Although the prone position was most commonly linked with pain, especially among women, our findings suggest that any sleeping position could potentially exacerbate pain in individuals with CLBP.

Conclusions

This study highlights the variability in how sleeping positions affect pain in patients with nonspecific CLBP. While the prone position is most frequently associated with increased pain, individual preferences and responses vary significantly, and often sidelying and supine positions provoke pain. The diversity in sleeping positions that exacerbate pain highlights the need for tailored advice in the management of patients with CLBP.

## Introduction

Chronic low back pain (CLBP) affects 10% to 23% of the working-age population, making it a prevalent health issue [1]. It leads to high treatment costs, increased sick leave, and significant individual suffering. More than half of those with CLBP report impaired sleep, often experiencing significantly worse sleep quality and efficiency than those without CLBP [2-5]. A one-point increase on a ten-point visual analog scale (VAS) correlates with a 10% higher likelihood of sleep disturbances [6]. Sleep’s duration and quality are crucial for physical and mental health [7]. In CLBP patients, poor sleep independently exacerbates medical costs and healthcare visits beyond the impacts of pain intensity and disability [8].

Significant associations exist between spinal pain and sleeping postures, including supine, prone, and partial side-lying positions [9]. Pain may arise from the lumbar spine’s tissues, such as the outer intervertebral discs, joint capsules, ligaments, fasciae, and muscles, due to prolonged low-load compression, shear, or torsion forces [10]. However, the relationship between sleeping positions and CLBP among patients seeking specialist care remains unexplored. This study aims to investigate the connection between nonspecific CLBP and pain related to certain sleeping positions. We examined how often different sleeping positions cause pain and their associations with low back pain (LBP), leg pain, and disability.

## Materials and methods

We conducted a cross-sectional study at the outpatient clinic of the Central Hospital of Central Finland’s spine department. Adults with LBP, unresponsive to conservative treatment in primary or occupational healthcare, were referred for further evaluation at this tertiary-care spine clinic following the LBP guideline [11]. We considered all referred patients as potential study participants. The Central Hospital, serving a population of 270,000, hosts the region’s only tertiary-care spine clinic. The inclusion criteria were Finnish-speaking adults over the age of 18, with LBP persisting for over three months. We excluded patients with radicular pain or specific CLBP causes like ankylosing spondylitis, fibromyalgia, tumors, and vertebral fractures.

The Research Ethics Committee of the Central Finland Health Care District approved the study (Approval Number 1E/2017). The procedure of the study was performed according to the relevant guidelines and regulations of the Declaration of Helsinki [12]. 

Study instrument

Patients completed a pre-visit questionnaire at home, detailing their medical history and main sleeping positions, including positions avoided due to LBP and factors worsening it. The questionnaire also gathered basic demographic information and back pain triggers and relievers. We assessed back and leg pain over the previous week using a VAS (scale 0-100) [13] and disability using the Oswestry Disability Index (ODI) [14]. The ODI measures daily life functionality across 10 areas, with scores ranging from 0 (the least disability) to 100 (the most severe disability). We used the validated Finnish ODI version [15]. 

Statistical analysis

We presented descriptive statistics as means ± standard deviation, counts (n), and percentages. We compared groups using the t-test, analysis of variance, and Pearson’s chi-squared test. We assessed variable normality with graphical methods and the Shapiro-Wilk W test. Data were analyzed using Stata 16.1 (StataCorp LP, College Station, TX, USA).

## Results

We included 375 consecutive adult patients with unspecific CLBP in the study. The average age of the patients was 51 ± 17 years, with females comprising 64% of the study population (n=240; Table 1). Eighty-five participants (23%) were smokers. The mean ODI score was 38% ± 18%, and the mean score on the VAS for pain was 63 ± 24. More than half of the patients reported referred leg pain, with mean VAS scores of 52 ± 66 for the right leg and 52 ± 33 for the left leg. Seventy-seven percent (n=289) of the patients experienced sleep disturbances due to LBP at night, and 87% (n=327) reported difficulties while getting up. A total of 92% (n=349) had challenges sleeping or rising in the morning because of LBP. We observed no significant differences between genders.

**Table 1:** Demographic and clinical data of patients (N=375) with chronic low back pain. Abbreviations: LBP: low back pain; SD: standard deviation; VAS: visual analog scale; ODI: Oswestry Disability Index. *Mean leg pain during last week in those who had leg pain.

Patient Information		Female (n=240)	Male (n=135)
Demographic data	Age (years), mean ± SD	52 ±17	50 ± 16
	Height (cm), mean ± SD	163 ± 7	179 ± 7
	Weight (kg), mean ± SD	74 ± 19	91 ± 20
	Body mass index (kg/m^2^), mean ± SD	28 ± 7	29 ± 6
Clinical data	Smoking, n (%)	50 (21%)	35 (26%)
	ODI, mean ± SD	39 ± 17	38 ± 19
	LBP (VAS), mean ± SD	64 ± 23	60 ± 25
	LBP at night, n (%)	194 (81%)	95 (70%)
	LBP in the morning, n (%)	212 (88%)	115 (85%)
	LBP night or morning, n (%)	225 (92%)	124 (92%)
	Leg pain right, n (%)	146 (61%)	76 (56%)
	Leg pain right (VAS), mean ± SD*	60 ± 25	56 ± 26
	Leg pain left, n (%)	140 (59%)	67 (50%)
	Leg pain left (VAS), mean ± SD*	58 ± 28	57 ± 26
Sleeping position	Side, n (%)	209 (87%)	118 (87%)
	Supine, n (%)	115 (48%)	61 (45%)
	Prone, n (%)	53 (22%)	29 (21%)

Side-lying was the predominant sleep position, with 87% (n=327) of both men and women preferring it (p<0.001). Approximately 47% of participants slept on their backs (n=176), and only 22% (n=82) preferred the prone position, again with no significant gender difference (p=0.69). Forty-three percent of the patients (n=161) reported changing their sleep position throughout the night. Individuals who slept in a prone position were, on average, 10 years younger than those who preferred other positions (Table 2), indicating a significant association between age and sleep position (p<0.001). Eight percent of patients (n=30) had a body mass index (BMI) between 35 and 40 kg/m^2^, and 5% (n=198) had a BMI over 40 kg/m^2^, with no significant correlation between BMI and sleep position (p=0.70). Patients who slept in a side-lying position had significantly higher mean ODI scores than those in other positions (p<0.001). However, we found no significant association between sleep position and LBP during the night (p=0.84).

**Table 2:** Demographic and clinical data of chronic low back pain patients (N=375) according to sleeping position. LBP: low back pain; SD: standard deviation; VAS: visual analog scale.

Patient data		Sleeping position			P-value
		Side (n=213)	Supine (n=114)	Prone (n=48)	
Demographic data	Female, n (%)	130 (61%)	83 (73%)	27 (56%)	0.69
	Male, n (%)	83 (39%)	31 (27%)	21 (44%)	
	Age in years, mean ± SD	54 ± 17	50 ± 16	42 ± 15	<0.001
	BMI (kg/m^2^), mean ± SD	28.4 ± 5.9	27.2 ± 6.0	28.8 ± 9.0	0.70
Clinical data	LBP (VAS), mean ± SD	63 ± 24	62 ± 24	60 ± 24	0.36
	Leg pain (VAS), mean ± SD	61 ± 28	52 ± 28	50 ± 30	0.007
	Oswestry, mean ± SD	41 ± 17	36 ± 17	33 ± 18	<0.001
Aggravates LBP	Standing, n (%)	151 (71%)	74 (65%)	24 (50%)	0.007
	Walking, n (%)	124 (58%)	61 (54%)	22 (46%)	0.11
	Sitting, n (%)	142 (67%)	72 (63%)	36 (75%)	0.54
	Working, n (%)	114 (54%)	67 (59%)	30 (63%)	0.19
	Leisure, n (%)	49 (23%)	24 (21%)	12 (25%)	0.94
	Lying, n (%)	49 (23%)	17 (15%)	12 (25%)	0.63
LBP occurs	Sleeping, n (%)	164 (77%)	87 (76%)	38 (79%)	0.84
	Getting up, n (%)	187 (88%)	99 (87%)	41 (85%)	0.64

**Figure 1:**
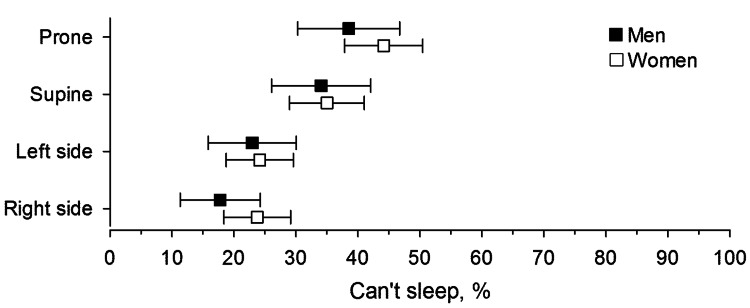
Sleeping position where patients with chronic low back pain were unable to sleep. Error bars indicate 95% confidence interval.

Seventy-six percent of the patients (n=285) reported avoiding one or more sleeping positions that worsened their LBP to the extent that they could not sleep in those positions (Figure 1). Both women and men were more likely to avoid sleeping in the prone and supine positions compared to the side-lying position (p<0.001). Forty-four percent of women (n=106), compared to 39% of men (n=53), were unable to sleep in the prone position, a difference that was statistically significant (p=0.04).

The supine position was avoided due to LBP by 34% of men (n=46) and 35% of women (n=84). Sixteen percent of women (n=38) and 13% of men (n=18) avoided lying on their left side due to LBP, while 16% of women (n=38) and 8% of men (n=11) avoided the right side. Only 8% of women (n=19) and 10% (n=14) of men reported avoiding both side-lying positions due to LBP. Fifty-six percent of the patients (n=210) reported that LBP was exacerbated at work, with no significant gender difference. Lying down relieved back pain for 59% of both men (n=80) and women (n=142).

## Discussion

Over 90% of the CLBP patients reported experiencing significant discomfort in bed at night and/or while getting up. Three-quarters identified certain sleeping positions as exacerbating their LBP. Prior research suggests that the prone position offers the least support for the lower back and is not recommended for individuals with CLBP [16]. Our findings support this perspective, highlighting the prone position as the most avoided posture for sleeping. Although body weight is more evenly distributed in the supine and prone positions, these positions may increase lumbar lordosis, subsequently loading the lumbar facet joints [17]. Moreover, the prone position is associated with significant issues such as buckling of the ligamentum flavum, increased spondylolisthesis, and facet joint subluxation leading to foraminal stenosis, as observed in magnetic resonance imaging in patients with low back pain with or without radiculopathy [18].

The side-lying posture is the most commonly adopted sleeping position, with over 60% of European adults preferring it for most of the night [19,20]. Similarly, our study found side-lying to be the preferred sleeping posture, possibly because other positions often aggravate LBP. However, the side-lying position was linked to a higher Oswestry Disability Index and more referred leg pain. This posture exerts considerable pressure on the hip area, potentially irritating the lower back [21].

Sleeping position showed no correlation with BMI or LBP, and gender did not significantly influence sleeping posture choice. However, we observed that older age groups were less likely to sleep in the prone position, aligning with previous research findings. LBP’s multifactorial nature includes potential contributions from environmental factors, hereditary factors, psychosocial aspects, degenerative disorders, or trauma, varying by individual [22]. Moreover, alcohol, drugs, smoking, and medicine abuse may influence both sleep and pain experiences. Sleep posture, as a factor, warrants further consideration. Notably, not all patients experienced pain while lying down; discomfort arose more commonly when getting out of bed, possibly due to deeper sleep, a higher pain tolerance, less severe pain, or biomechanical factors related to movement during sleep. Cary et al. [20] discovered that patients with LBP spent significantly more time in postures that provoked their symptoms compared to asymptomatic controls. Although the sample sizes were small, the significance of advising on sleeping position in preventing and treating LBP is supported by randomized controlled studies [23,24]. In those studies, a significant majority of participants reported that certain sleeping positions adversely affected their LBP, necessitating the avoidance of these sleeping positions. Interestingly, this contrasts with this study’s findings, which state that over half of the patients felt relief from CLBP when lying down. This suggests that a lower axial load on the spine while lying down and finding a comfortable position can alleviate CLBP. However, sleep positions may change uncontrollably multiple times throughout the night, and thus, pain-provoking sleeping positions may exist [20].

The strength of this study lies in its large sample size, which represents patients with moderate to severe CLBP seeking specialist consultation. Employing the VAS and ODI to assess pain intensity and disability provides data about the severity of symptoms in the patient population. The results offer a broad perspective on CLBP and sleeping positions among a clinic-attending population seeking help for their symptoms. The study highlights both variations in sleeping positions and their avoidance due to pain, offering a nuanced understanding of how CLBP affects this part of daily living.

The study had some limitations. The study relied on patient-reported sleep positions, capturing only the positions at sleep onset and upon awakenings, thus not fully accounting for changes throughout the night. Adults change their sleeping position up to four times per hour in their twenties, decreasing to twice per hour after age 65 [25]. Including objective measurements to track sleeping position changes throughout the night would have added valuable insights. The cross-sectional design of this study limits our ability to establish causality between sleeping positions and CLBP severity.

## Conclusions

This study explored the preferred sleeping position and the frequency of increased pain in various sleeping positions among patients with nonspecific CLBP. It examined the relationship between sleeping positions, pain, and disability levels. The side-lying posture was the most preferred sleeping position. Our findings indicate that specific sleeping positions aggravate LBP in a majority of CLBP patients. While prone and supine positions are most likely to provoke LBP, side-lying positions also cause discomfort for many. The variation in pain-inducing sleeping positions among individuals underscores the importance of personalized guidance in managing CLBP.
